# Contrasting Effects of Chronic Glucokinase Activation and Inhibition on Pancreatic Beta‐Cell Function

**DOI:** 10.1096/fj.202504049R

**Published:** 2026-03-27

**Authors:** Matthew Lloyd, Natascia Vedovato, Samuel J. Mullan, Alice Elphick, Aerin J. Dumenil, Chantal Rufer, Alexandra C. Title, Burcak Yesildag, Elizabeth Haythorne, Frances M. Ashcroft

**Affiliations:** ^1^ Department of Physiology, Anatomy and Genetics University of Oxford Oxford UK; ^2^ Institute for Neuroscience and Cardiovascular Research University of Edinburgh, The Queen's Medical Research Institute Edinburgh UK; ^3^ InSphero AG Schlieren Switzerland

**Keywords:** beta‐cell, diabetes, dorzagliatin, glucokinase, glucotoxicity, hyperglycaemia, insulin secretion

## Abstract

Chronic hyperglycaemia impairs insulin secretion by disrupting beta‐cell metabolism and reducing expression of insulin. These changes are mediated by glucose metabolites downstream of glucokinase. We hypothesized that chronically enhancing glucokinase activity at low glucose would mimic the effects of glucotoxicity and, conversely, that reducing glucokinase activity would prevent the deleterious effects of chronic hyperglycaemia. Human islet microtissues or INS‐1 cells were cultured at low or high glucose for 2 weeks (islets) or 48 h (INS‐1 cells) in the presence or absence of either a glucokinase activator (MK‐0941 or dorzagliatin) or a glucokinase inhibitor (mannoheptulose). We measured changes in glucose‐stimulated insulin secretion, insulin content, glycogen content, gene expression, and oxygen consumption. Chronic glucokinase activation in low‐glucose‐cultured islet microtissues and INS‐1 cells downregulated insulin secretion and content, impaired mitochondrial metabolism, and altered metabolic gene expression, to the same extent as high‐glucose culture. Conversely, reduction of glucokinase activity in high‐glucose‐cultured islet microtissues by mannoheptulose prevented the changes produced by chronic hyperglycaemia. Moreover, the effects of chronic hyperglycaemia on insulin secretion and metabolic gene expression (but not insulin content) were largely reversed by subsequent inhibition of glucokinase. Chronic glucokinase activation in euglycaemia thus mimics the effects of chronic hyperglycaemia, whereas partial inhibition of glucokinase in hyperglycaemia restores normal beta‐cell function. This explains why, in the long term, glucokinase activators are ineffective at controlling glycaemia in type 2 diabetes, and demonstrates that normalizing glucokinase activity can prevent and reverse chronic glucose toxicity.

## Graphical abstract

In the non‐diabetic beta‐cell (top left) activation of glucokinase (bottom left) leads to inhibition of mitochondrial metabolism and thereby impairs insulin secretion. The effect is similar to that of chronic hyperglycaemia as seen in the diabetic beta‐cell (top right). Partial inhibition of glucokinase in the diabetic beta‐cell can restore mitochondrial metabolism and insulin secretion (bottom right).

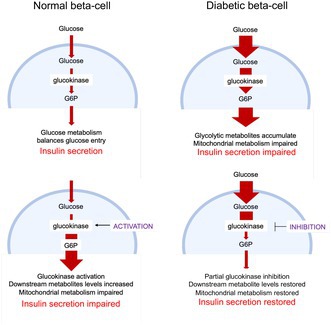

**Abbreviations**
ADP: adenosine diphosphate
Aldob: aldolase B
ATP: adenosine triphosphate
Eno1: enolase 1
Gapdh: glyceraldehyde‐3‐phosphate dehydrogenase
GCK: glucokinase
GCK‐MODY: maturity‐onset diabetes of the young due to inactivation of GCK
GKA: glucokinase activator
HG‐cells: high‐glucose cultured cells
HI: congenital hyperinsulinism of infancy
Hprt1: hypoxanthine phosphoribosyltransferase 1
Hspa8: heat shock protein family A (Hsp70) member 8
Idh2: isocitrate dehydrogenase
Ins1: insulin 1
Ins2: insulin 2
LG‐cells: low‐glucose cultured cells
Mafa: MAF bZIP transcription factor A
Mdh2: malate dehydrogenase
Ndufa4: NDUFA4 mitochondrial complex associated
Ndufs2: NADH:ubiquinone oxidoreductase core subunit S2
Ndufs8: NADH:ubiquinone oxidoreductase core subunit S8
Neurod1: neuronal differentiation 1
Nkx6‐1: NK6 homeobox 1
Pax6: paired box 1
Pdk1: pyruvate dehydrogenase kinase 1
Pdx1: pancreatic and duodenal homeobox 1
Pfkfb2: 6‐Phosphofructo‐2‐Kinase/Fructose‐2,6‐Biphosphatase 2
Pfkfb3: 6‐phosphofructo‐2‐kinase/fructose‐2,6‐biphosphatase 3
Pfkl: phosphofructokinase (liver type)
Sdha: succinate dehydrogenase complex flavoprotein subunit A
T2D: type 2 diabetes

## 1 Introduction

Glucokinase is a key regulatory enzyme in the pancreatic beta‐cell and plays a crucial role in glucose‐stimulated insulin secretion [1]. It serves as the beta‐cell glucose sensor, enabling insulin secretion to be matched to the blood glucose concentration. An increase in circulating blood glucose stimulates glucose uptake and metabolism by the beta‐cell, leading to an increase in cytosolic ATP and a fall in cytosolic MgADP [2]. These changes in adenine nucleotides cause inhibition of the ATP‐sensitive potassium (K_ATP_) channel, which triggers membrane depolarisation and leads to the opening of voltage‐gated calcium channels, calcium influx, and exocytosis of insulin granules [2].

The initial step in glucose metabolism is phosphorylation of the sugar, which in beta‐cells is mediated by the enzyme glucokinase [1]. Glucokinase differs from other hexokinases due to several unique properties that make it an ideal glucose sensor [1]. It has a sigmoidal glucose dependency, with an inflection point at ~4 mM glucose, close to the threshold for insulin secretion (~5 mM in human beta‐cells and slightly higher in mice). Uniquely among hexokinases, it has a high Km for glucose (8 mM in murine islets and 4 mM in human islets); it does not saturate at physiological glucose concentrations (Vmax > 20 mM), and it is not inhibited by physiological concentrations of glucose‐6‐phosphate. These properties ensure that the rate of glucose phosphorylation is proportional to the extracellular glucose concentration across the entire physiological range (3–15 mM). Glucokinase expression is restricted to glucose‐sensing cells, which include beta‐cells, some neuroendocrine and entero‐endocrine cells, certain glucose‐sensing neurons, and hepatocytes. Most cells express hexokinases 1–3, which have a high affinity for glucose, saturate at low glucose concentrations, and thus are insensitive to changes in glucose over the physiological range [3].

Given its central role in insulin release, it is not surprising that mutations in glucokinase can cause disorders of insulin secretion [4]. Homozygous loss‐of‐function mutations cause permanent neonatal diabetes [5], whereas heterozygous loss‐of‐function mutations produce maturity onset diabetes of the young (GCK‐MODY) [6]. The latter is characterized by mild fasting hyperglycaemia and blood glucose levels are simply regulated around this new set point (usually 5.5 to 8 mM glucose). GCK‐MODY does not require therapy and is not associated with progressive beta‐cell decline or the development of secondary complications [7, 8, 9]. Activating glucokinase mutations cause congenital hyperinsulinism of infancy (HI) [10, 11]. They often act by increasing the affinity of the enzyme for glucose and this leads to inappropriate and unregulated insulin secretion at low blood glucose levels. Although a few mutations cause very severe life‐threatening hypoglycaemia [12], many result in a milder phenotype that is responsive to drug therapy and may even be asymptomatic [11]. The critical importance of glucokinase for beta‐cell glucose sensing is also illustrated by the fact that aberrant expression of hexokinase 1 in beta‐cells results in unregulated insulin secretion and hypoglycaemia [13].

The increasing prevalence of type 2 diabetes (T2D) worldwide has stimulated the search for therapies that can prevent the progressive decline in beta‐cell function that characterizes the disease and reduce the accompanying increase in blood glucose that leads to debilitating secondary complications. Glucokinase activators (GKAs) have been extensively studied as possible therapeutics in T2D, the aim being to boost glucose metabolism and increase insulin secretion and hepatic glycogen synthesis [14]. However, to date, GKAs have largely proved disappointing. In particular, the effects of GKAs on glycaemic control in patients with T2D were transitory: no long‐term improvements were found [15, 16]. The incidence of hypoglycaemia increased and triglycerides were also elevated. More recently, dorzagliatin has been approved for use in China, either alone or in combination with metformin [17, 18]. However, clinical trials showed only modest reductions in HbA1c after 52 weeks [17, 18, 19, 20], HbA1c was still not normalised, and longer‐term efficacy, or efficacy in populations outside China, is still to be established.

One possible explanation for the failure of many GKAs is that, by enhancing glycolytic flux, they may exacerbate the effects of hyperglycaemia, leading to further beta‐cell decline. Many studies have shown that chronic hyperglycaemia, such as occurs in diabetes, leads to changes in beta‐cell gene expression that reduce mitochondrial metabolism, ATP production, insulin content, and ultimately insulin secretion [21, 22, 23, 24, 25]. GAPDH activity is markedly reduced (despite increased protein expression), leading to a bottleneck in glycolytic metabolism [26]. Glycogen accumulation also occurs [27, 28]. Beta‐cells have low levels of lactate dehydrogenase activity, limiting the amount of NADH that can be re‐oxidised to NAD^+^ for utilization as a cofactor by GAPDH [29, 30]. Together with the downregulation of glucose‐6‐phosphatase [25], this results in excess glucose entering the beta‐cell that cannot be metabolised being stored as glycogen.

Studies in diabetic mouse islets, or in beta‐cell lines, have indicated that changes in gene expression and metabolism which occur in response to chronic hyperglycaemia are mediated not by glucose itself, but by a glycolytic metabolite that lies downstream of glucokinase [21, 26], and most of the adverse effects of hyperglycaemia on beta‐cell function can be prevented by reducing flux through glucokinase [21, 26, 31, 32]. In particular, mannoheptulose, a competitive inhibitor of glucokinase [33], prevented the changes in gene expression, mitochondrial metabolism, insulin content and glucose‐stimulated insulin release induced by chronic hyperglycaemia [21, 26, 31]. Furthermore, in diabetic *db/db* mice, glucokinase haploinsufficiency ameliorated glucose intolerance and glucose‐stimulated insulin secretion [32]. Βeta‐cell mass and insulin content were also increased. Similarly, in a mouse model with an inducible Kir6.2 mutation, glucokinase haploinsufficiency increased beta‐cell mass, insulin gene expression and content, and improved plasma glucose and glucose tolerance [34]. These studies suggest that by reducing flux through glucokinase the deleterious effects of chronic hyperglycaemia can be ameliorated [35, 36, 37, 38]. This raises the question of whether chronic glucokinase activation might have adverse effects on beta‐cell function by *increasing* glycolytic flux. Most studies to date have shown that GKAs acutely enhance insulin release. However, given that acute exposure to elevated glucose increases secretion [2] but that long‐term exposure decreases secretion [21, 26, 32], we sought to determine if chronic exposure to GKAs might impair insulin release.

## 2 Materials and Methods

### 2.1 Human Islet Microtissues

Human islet microtissues produced by hanging‐drop‐based scaffold‐free reaggregation of dispersed primary non‐diabetic human islets [39] were generated by InSphero (InSphero AG, Schlieren, Switzerland) using cadaveric organs from the USA. Donor information is given in Supplementary Table 1. In activator experiments, human islet microtissues were maintained in 3D InSight Human Islet Maintenance Medium (InSphero AG), containing 5.5 mM glucose (with or without 0.1 μM MK‐0941) or 16.7 mM glucose. In inhibitor experiments, human islet microtissues were maintained in 3D InSight Human Islet Maintenance Medium (InSphero AG), containing 5.5 mM or 16.7 mM glucose, with or without mannoheptulose (4 mM or 8 mM), as indicated, for 14 days. In a few experiments, human islet microtissues were maintained in 3D InSight Human Islet Maintenance Medium containing 16.7 mM glucose for 7 days, followed by 7 days culture in either 5.5 mM glucose or 16.7 mM glucose with mannoheptulose (4 mM or 8 mM). The medium and compounds were renewed every 2–3 days.

### 2.2 INS‐1 Cells

INS‐1 (832/13) cells (abbreviated here as INS‐1 cells) were originally developed by Claes Wollheim (Geneva) and supplied by Patrik Rorsman (Oxford). They were negative for mycoplasma contamination. They were cultured in RPMI‐1640 medium supplemented with 10% FBS, 1% Pen/Strep, 50 μM β‐mercaptoethanol, 1 mM sodium pyruvate, 10 mM HEPES, and 2 mM L‐glutamine (standard culture medium; all Sigma‐Aldrich) in a humidified atmosphere of 5% CO_2_ / 95% air at 37°C. Unless otherwise stated, the glucose concentration was 11 mM. In order to replicate normoglycaemic (control) and hyperglycaemic (diabetic) conditions in vitro, INS‐1 cells were subsequently cultured at low glucose (5 mM; LG‐cells) or high glucose (16.7/25 mM; HG‐cells) for 48 h prior to experiment. For activator studies, cells were cultured at 25 mM glucose for 48 h; or at 5 mM glucose with either the glucokinase activators MK‐0941 (10 μM) or dorzagliatin (1 μM), or with the appropriate vehicle control (DMSO 0.1%), for 48 h. These were the lowest GKA concentrations that increased glucokinase activity in LG‐cells to a level equivalent to that observed in HG‐cells (Figure S1A,B). For inhibitor prevention studies, cells were cultured at 5 mM or 16.7 mM glucose for 48 h either with or without mannoheptulose for 48 h. Mannoheptulose is a pan‐hexokinase inhibitor but is functionally a glucokinase inhibitor in beta‐cells due to the dominance of GCK over other isoforms (discussed in section 3.1). Control studies indicated that 4 mM mannoheptulose reduced glucokinase activity in cells cultured at 16.7 mM glucose to that found at low glucose (Figure S2). For reversal experiments, cells were cultured at 5 mM glucose for 48 h or at 16.7 mM glucose for 48 h without mannoheptulose followed by 48 h at 16.7 mM glucose with mannoheptulose.

### 2.3 Hexokinase Activity

Glucokinase activity was quantified in cell lysates using a colorimetric hexokinase assay kit (Abcam ab136957), according to the manufacturer's instructions. Enzyme activity was normalised to the protein content of the well, as determined by BCA assay (Thermo Scientific).

### 2.4 Glucose‐Stimulated Insulin Secretion (GSIS)

Human islet microtissues were washed twice with 70 μL buffer containing (in mM) NaCl 131, KCl 4.8, CaCl_2_ 1.3, Hepes 25, KH_2_PO_4_ 1.2, 1 MgSO_4_ 1.2, BSA 0.5% plus 2.8 mM glucose and equilibrated for 1 h in the same solution. GSIS was performed in the Akura 96 plate (InSphero) in 50 μL KRHB containing different glucose concentrations for 2 h. The supernatant was collected for ELISA analysis (STELLUX Chemi Human Insulin ELISAs, ALPCO). After GSIS, the microtissues were lysed to analyse the total insulin content. Insulin values are expressed as ng of insulin per islet microtissue.

INS‐1 (832/13) cells were cultured in RPMI‐1640 medium containing low (5 mM) or high (16.7 or 25 mM) glucose with or without a glucokinase activator or inhibitor, as indicated for 48 h. On the day of the assay, cells were washed twice in Krebs–Ringer‐bicarbonate buffer containing (in mM): 140 NaCl, 3 KCl, 0.5 NaH_2_PO_4_, 2 NaHCO_3_ [saturated with CO_2_], 1.5 CaCl_2_, 0.5 MgSO_4_, 10 HEPES (pH 7.4) and 0.1% (w/v) fatty acid‐free (FFA) BSA. Cells were pre‐incubated with 2 mM glucose Krebs buffer at 37°C for 60 min, after which the buffer was removed, and cells were incubated with Krebs buffer containing 2 mM or 20 mM glucose for 30 min. The supernatant was removed and cells harvested either in acid ethanol (for total insulin content) or in RIPA lysis buffer (for protein content). Insulin levels in the supernatant and cell lysates were determined by insulin ELISA (Mercodia, Uppsala, Sweden). Insulin secretion and insulin content were normalised to the protein content of the well. Unless otherwise stated, glucokinase activators and inactivators were removed before starting the insulin secretion assay.

### 2.5 Gene Expression

#### 2.5.1 qPCR (INS‐1 Cells)

Total RNA was isolated using the ReliaPrep RNA Cell Miniprep System (Promega), according to the manufacturer's instructions. RNA concentration was determined using a NanoDrop ND‐1000 spectrophotometer (Thermo Scientific) and RNA reverse transcribed using Applied Biosystems High‐Capacity cDNA Transcription Kit (ThermoFisher). Quantitative PCR was performed using TaqMan probes or custom primers (Supplementary Table 2) and the Applied Biosystems StepOne Plus Real‐Time PCR system (Applied Biosystems). All reactions were performed in duplicate or triplicate. Data were quantified according to the delta–delta Ct method with normalisation to the housekeeping genes *Hprt1* and *Hspa8* or *Actb*.

#### 2.5.2 RNA‐Sequencing (Human Islet Microtissues)

Transcriptomic analysis of hexokinase isoforms (GCK, HK1, HK2, and HK3) in human islet microtissues was performed using detector oligo–based targeted RNA sequencing assay (TempO‐Seq), as previously described [40]. In short, following 7‐day culture in Human Islet Maintenance Media (InSphero) containing 5.5 mM or 16.7 mM glucose, three microtissues were pooled, washed once in 70 μL PBS without Ca^2+^/Mg^2+^ (Sigma‐Aldrich) and lysed in 15 μL of 1× Enhanced Lysis Buffer (BioSpyder Technologies). For each condition, five replicates were sequenced. Sequencing libraries were generated relying on the standard TempO‐Seq chemistry (Human Whole Transcriptome 2.0; 22 537 p robes targeting 19 701 genes), and sequencing was performed by BioSpyder. Reads were processed using TempO‐SeqR program (BioSpyder Technologies), probe counts were collapsed to gene‐level counts, and genes with zero counts across all samples were removed. Differential expression analysis was performed using DESeq2, batch effects were corrected with surrogate variable analysis, and *p*‐values were adjusted using the Benjamini–Hochberg method (FDR < 0.001).

### 2.6 Glycogen Measurements

INS‐1 cells were cultured at 5 mM (low glucose, LG‐cells) or 25 mM (high glucose, HG‐cells) glucose for 48 h. To help reduce background glucose contamination, cells were transferred to media containing 5 mM glucose for 30 min prior to sample collection. They were then washed twice with ice‐cold PBS and lysed by sonication in ultrapure water. Lysates were immediately boiled for 10 min, centrifuged to pellet debris, and the glycogen content of the supernatant was determined using the Glycogen Colorimetric/Fluorometric Assay Kit (Biovision K646) as per the manufacturer's instructions. The protein concentration in the supernatant was determined by BCA assay; glycogen content is expressed as μg per mg protein.

### 2.7 Respirometry

The Seahorse XFe24 Extracellular Flux Analyser (Seahorse Bioscience, Copenhagen, Denmark) was used to assess a range of metabolic parameters by real‐time monitoring of cellular oxygen consumption rate (OCR), as described [25]. In brief, INS‐1 cells were cultured at various glucose concentrations with or without MK‐0941 or mannoheptulose, as stated above, for 48 h and washed in serum‐free unbuffered RPMI medium (Agilent) containing 2 mM glucose for 1 h prior to measurement. Glucose‐stimulated respiration was measured by addition of 20 mM glucose. Mitochondrial efficiency was assessed using compounds that inhibit specific mitochondrial processes: ATP‐linked respiration (oligomycin) and proton leak (antimycin A + rotenone). Data are presented as either pmol O_2_/min/μg protein or were normalised to the last baseline measurement prior to the addition of 20 mM glucose (100%). The % change in OCR following the addition of a compound/substrate was also calculated.

### 2.8 Chemicals

MK‐0941 was obtained from Cayman Chemical and dorzagliatin from Stratech. All other chemicals came from Sigma.

### 2.9 Statistical Analysis

Unless otherwise stated, results are presented as individual data points and mean ± s.e.m. INS‐1 cell experiments had 3 or more technical replicates and the mean value of all replicates was taken as *n* = 1 experiment. For human islet microtissues, *n* = 6 technical replicates were performed using islets from the same donor and the mean for each donor is presented; full data from individual donors are shown in separate Figures S1–S6. Significance was tested using one‐way or two‐way ANOVA with Bonferroni's multiple comparisons test, using GraphPad Prism software. Differences were considered statistically significant if *p* < 0.05.

## 3 Results

### 3.1 Effects of Glucokinase Activation

**FIGURE 1:**
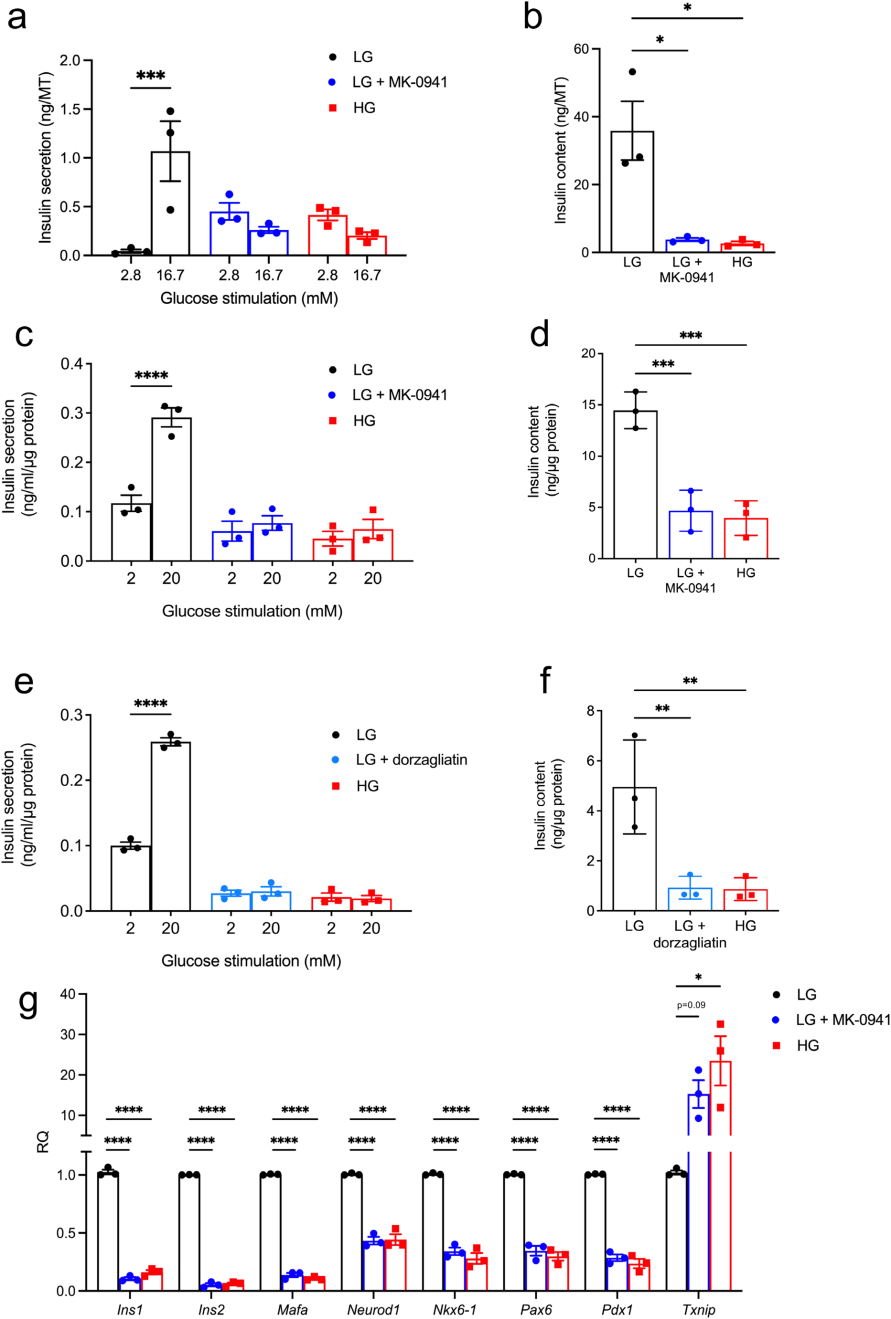
Effects of glucokinase activation on insulin secretion and insulin content. (a, b) Insulin secretion at 2.8 mM and 16.7 mM glucose (a) and insulin content (b) in human islet microtissues cultured at low glucose (LG: 5.5 mM glucose), LG + 0.1 μM MK‐0941, and high glucose (HG: 16.7 mM) glucose for 14 days (*n* = 3 donors, 6 replicates/donor). (c, d) Insulin secretion at 2 and 20 mM glucose (c) and insulin content (d) in INS‐1 cells cultured at 5 mM glucose (LG), 5 mM glucose + 10 μM MK‐0941 (LG + MK‐0941), or 25 mM glucose (HG) for 48 h (*n* = 3 biologically independent experiments). (e, f) Insulin secretion at 2 and 20 mM glucose (e) and insulin content (f) in INS‐1 cells cultured at 5 mM glucose (LG), 5 mM glucose + 1 μM dorzagliatin (LG + dorzagliatin), or 25 mM glucose (HG) for 48 h (*n* = 3 biologically independent experiments). (g) mRNA levels of the indicated genes in INS‐1 cells cultured at 5 mM glucose (LG), 5 mM glucose + 10 μM MK‐0941 (LG + MK‐0941), or 25 mM glucose (HG) for 48 h. (*n* = 3 biologically independent experiments) All panels show individual data points and mean ± s.e.m. *p < 0.05, **p < 0.01, ***p < 0.001, ****p < 10^−4^. One‐way (b, d, f, g) or two‐way (a, c, e) ANOVA with Bonferroni's multiple comparisons test.

We investigated the effects of chronic glucokinase activation on insulin secretion from human islet microtissues cultured for 14 days at low (5.5 mM) glucose. When cultured at high (16.7mM) glucose this model exhibits a robust and physiologically relevant glucotoxic phenotype [39] which is comparable to that seen in studies of isolated islets from 2‐week‐diabetic mice [25, 26, 27]. Figure 1a shows that chronic exposure to the selective glucokinase activator MK‐0941 (0.1 μM) increased basal insulin secretion in microtissues exposed acutely to 2.8 mM glucose, but reduced stimulated secretion at 16.7 mM glucose. It also markedly reduced insulin content (Figure 1b). The results were similar to those caused by chronic culture at 16.7 mM glucose. Results from each individual donor are presented in the Supporting Information (Figure S3–5). These data argue that enhanced glycolytic flux, whether due to increased glucokinase activity or to elevated extracellular glucose, impairs insulin secretion from human beta‐cells.

We further examined the effects of chronic glucokinase activation in the insulin‐secreting beta‐cell line INS‐1 832/13 (INS‐1 cells), using the selective activators MK‐0941 [15, 16] and dorzagliatin [17, 18, 19, 20].

We determined the lowest concentration of each activator that increased glucokinase activity in LG‐cells to a level equivalent to that observed in HG‐cells (Figure S1A,B). To demonstrate the validity of measuring total hexokinase activity as a readout of glucokinase activation, we compared expression of *Gck* and *Hk1* in INS‐1 cells (by qPCR) or *Gck* and *HK1‐3* in human islet microtissues (by transcriptomic analysis) following chronic culture under low or high glucose conditions. We found that *GCK* expression dominated in islet microtissues at both high and low glucose (Table S3). *Gck* expression fell in HG‐cells (Figure S1C) but, given the very low level of *Hk1* expression in INS‐1 cells (undetectable in some datasets [41]), would remain the dominant isoform. These findings are consistent with the “disallowed gene” status of *HK1‐3* in islets and align with our previous findings in diabetic mouse models, where *Gck* remained over 20‐fold more abundant than other hexokinase isoforms in diabetic islets [25]. We conclude that the contribution of low‐Km isoforms to the measured hexokinase activity is minimal and that total hexokinase activity therefore provides a valid measure of glucokinase activity in beta‐cells.

We first tested the effects of chronic glucokinase activation by MK‐0941 and dorzagliatin on insulin secretion in INS‐1 cells cultured at low glucose. Forty‐eight hours exposure to MK‐0941 produced a dramatic reduction in glucose‐stimulated insulin secretion and insulin content, comparable to that caused by chronic culture at 25 mM glucose (Figure 1c,d). A similar result was found with dorzagliatin (Figure 1e,f). These data are consistent with our findings from human islet microtissues and demonstrate that the effects of chronic glucokinase activation are consistent across different GKAs.

The marked decrease in insulin content produced by MK‐0941 or chronic hyperglycaemia (Figure 1d) was accompanied by a reduction in expression of the insulin genes *Ins1* and *Ins2* and the insulin gene transcription factors *Mafa, Neurod1, Nkx6‐1, Pax6*, and *Pdx1* (Figure 1g). Furthermore, *Txnip*, which inhibits insulin production via inducing expression of microRNA miR‐204, leading to downregulation of *Mafa* [42], was markedly upregulated both by 25 mM glucose and by glucokinase activation at 5 mM glucose (Figure 1g). Thus, the lower insulin content appears to be a consequence of reduced insulin gene expression.

**FIGURE 2:**
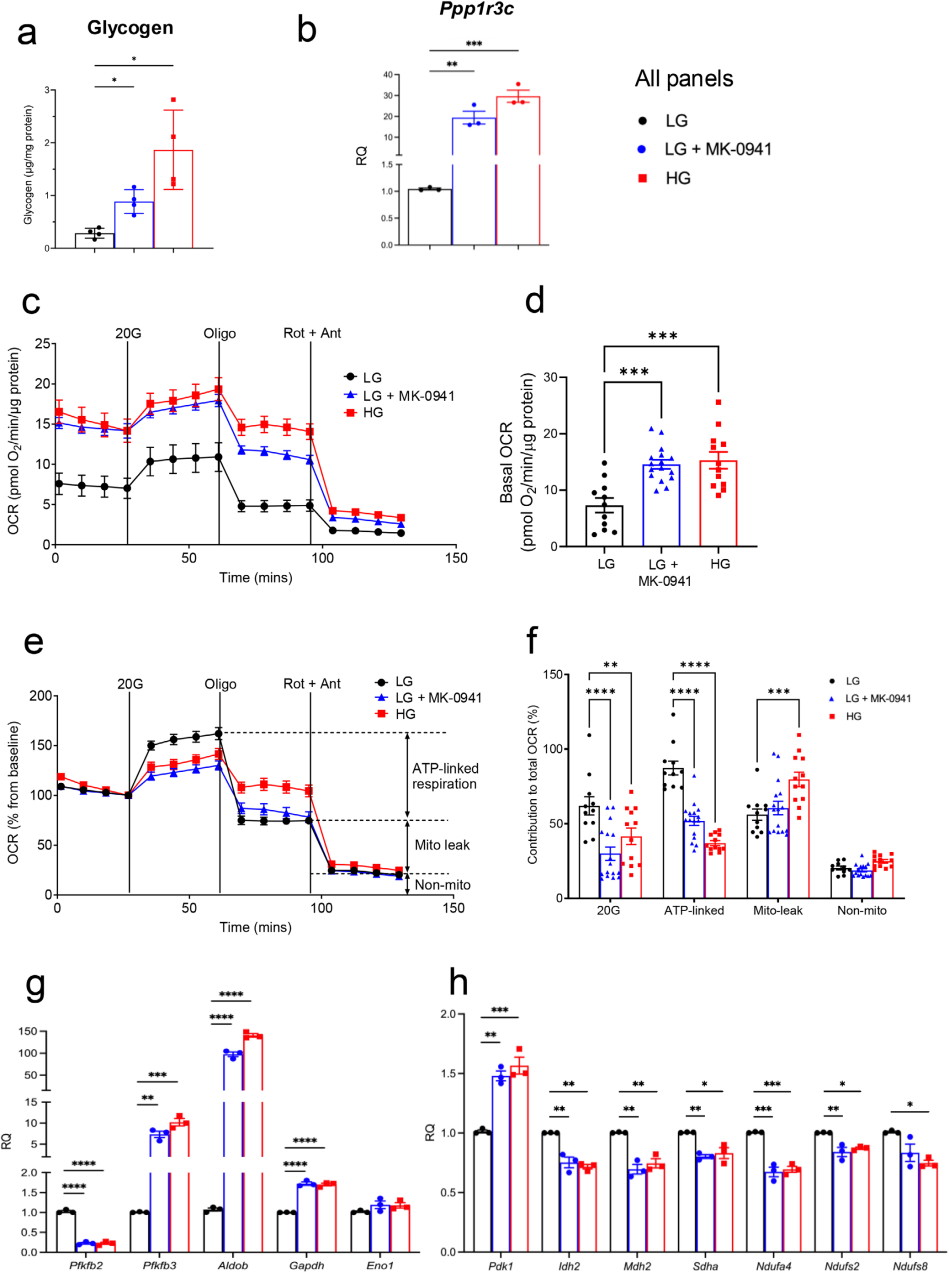
Chronic glucokinase activation impairs oxidative metabolism. (a, b). Glycogen content (a) (*n* = 3 biologically independent experiments) and mRNA levels of *Ppp1r3c* (b) (*n* = 4 biologically independent experiments) in INS‐1 cells cultured at 5 mM glucose (LG‐cells), 5 mM glucose + 10 μM MK‐0941 (LG + MK‐0941), or 25 mM glucose (HG‐cells) for 48 h. (c) OCR of INS‐1 cells cultured for 48 h at 5 mM glucose (LG), 5 mM glucose + 10 μM MK‐0941 (LG + MK‐0941) or 25 mM glucose (HG). OCR was first measured at 2 mM glucose and then after sequential addition of 20 mM glucose (20G), 1 μM oligomycin (Oligo), and 0.5 μM rotenone + 0.5 μM antimycin A (Rot + Ant) (LG, *n* = 11; HG, *n* = 12; LG + MK‐0941, *n* = 16 biologically independent experiments). (d) Basal OCR at 2 mM glucose. Same data as in (c). (e) OCR expressed as the percentage change from baseline (at 2 mM glucose) and after sequential addition of 20 mM glucose (20G), 1 μM oligomycin (Oligo), and 0.5 μM rotenone + 0.5 μM antimycin A (Rot + Ant) (*n* = 11 LG, 12 HG, and 16 LG + MK‐0941 biologically independent experiments). (f) Percentage change in OCR when glucose was raised from 2 mM to 20 mM (20G), after oligomycin was applied (ATP‐linked), after rotenone and antimycin A were applied (mito‐leak), and remaining non‐mitochondrial OCR (non‐mito). Same data as in (e) (g, h) mRNA levels of the indicated glycolytic (g) and mitochondrial (h) genes in INS‐1 cells cultured at 5 mM glucose (LG), 5 mM glucose + 10 μM MK‐0941 (LG + MK‐0941), or 25 mM glucose (HG) for 48 h (*n* = 3 biologically independent experiments). All panels show individual data points and mean ± s. e. m. *p < 0.05, **p < 0.01, ***p < 0.001, ****p < 0.0001. Welch's ANOVA with Dunnett's correction (a), one‐way ANOVA with Bonferroni's multiple comparisons test (b, d, g, h), or two‐way ANOVA with Bonferroni's multiple comparisons test (f).

Both chronic glucokinase activation and chronic hyperglycaemia increased glycogen content (Figure 2a) and expression of *Ppp1r3c*, a glycogen‐targeting subunit of protein phosphatase 1 that strongly promotes glycogen synthesis in beta‐cells [43] (Figure 2b). Glycogen is not normally found in significant quantities in beta‐cells [27, 28, 43] and its production suggests that beta‐cell metabolism is impaired by chronic glucokinase activation, as previously reported for beta‐cells exposed to chronic hyperglycaemia [25, 26]. The presence of glycogen stores may contribute to the elevated basal insulin secretion observed in human islet microtissues (Figure 1a) by providing a fuel source that can be mobilised when extracellular glucose is low.

To confirm that metabolism is impaired, we measured the effect of MK‐0941 on basal (at 2 mM glucose) and stimulated (20 mM) oxygen consumption (OCR) (Figure 2c–f). Basal OCR was elevated in INS‐1 cells cultured for 48 h at 5 mM glucose plus MK‐0941 to the same extent as in cells cultured at 25 mM glucose (Figure 2c,d). Furthermore, the glucokinase activator reduced both glucose‐stimulated OCR and ATP‐linked respiration in LG‐cells to the same extent as culture at 25 mM glucose (Figure 2e,f). However, whereas mitochondrial leak increased in HG‐cells, it was unaffected by chronic glucokinase activation in LG‐cells.

Consistent with the reduction in glucose‐stimulated oxidative metabolism, expression of numerous metabolic genes was affected by glucokinase activation (Figure 2g,h). For example, the glycolytic genes *Aldob*, *Pfkfb3*, and *Gapdh* were upregulated, while *Pfkfb2* was downregulated. Genes encoding the TCA cycle enzymes *Idh2, Mdh2*, and *Sdha*, as well as various electron transport chain proteins (*Ndufs2, Ndufs8*, and *Ndufa4*), were all downregulated. In addition, pyruvate dehydrogenase kinase 1 (Pdk1), which phosphorylates and inhibits pyruvate dehydrogenase, and thus substrate entry into the TCA cycle, was upregulated. These changes are similar to those observed in INS‐1 cells exposed to chronic hyperglycaemia or in mouse and human islets in response to diabetes [25, 26, 44, 45, 46, 47].

Together, these data suggest that chronic glucokinase activation in normoglycaemia has multiple adverse effects on pancreatic beta‐cells, resembling those produced by chronic hyperglycaemia and diabetes.

### 3.2 Effects of Glucokinase Inactivation

**FIGURE 3:**
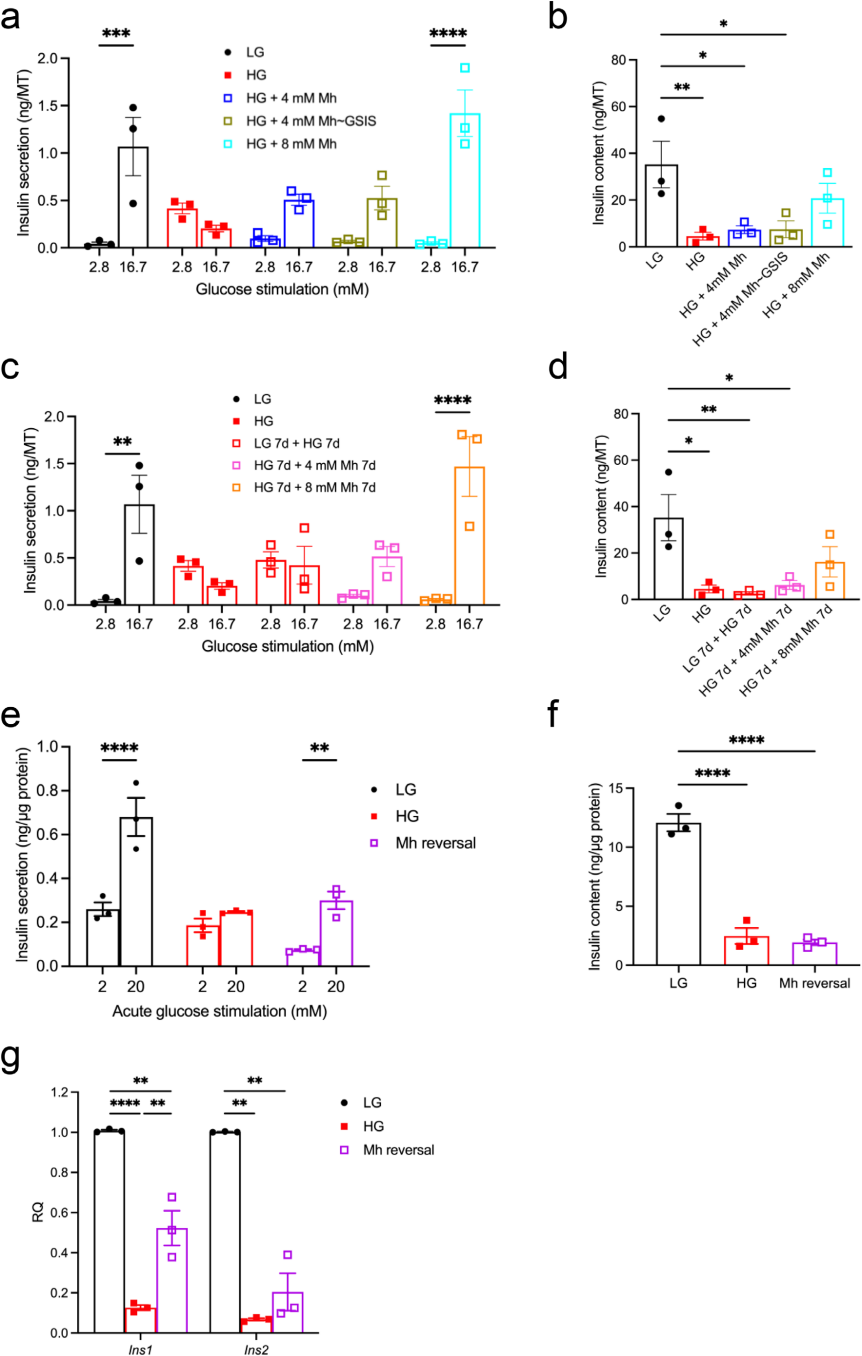
Partial glucokinase inhibition prevents and reverses the effects of chronic hyperglycaemia. (a, b) Insulin secretion at 2.8 mM and 16.7 mM glucose (a) and insulin content (b) in human islet microtissues cultured for 14 days at LG (black circles), HG (filled red squares), and HG + 4 mM (open dark blue and olive squares) or 8 mM (open light blue squares) mannoheptulose (Mh). The drug was omitted from the assay medium for all experiments except for the data shown in olive (HG + 4 mM Mh ~ GSIS). *n* = 3 donors, 6 replicates/donor. (c, d) Insulin secretion at 2.8 mM and 16.7 mM glucose (c) and insulin content (d) in human islet microtissues cultured for 14 days at LG (black circles), 14 days at HG (filled red squares), 7 days at LG followed by 7 days at HG (open red squares), 7 days at HG followed by 7 days at HG + 4 mM Mh (magenta squares), 7 days at HG followed by 7 days at HG + 8 mM Mh (open orange squares). The drug was removed from the assay medium for all experiments. *n* = 3 donors, 6 replicates/donor. (e, f). Insulin secretion at 2 and 20 mM glucose (e) and insulin content (f) in INS‐1 cells cultured at 5 mM glucose (LG) or 16.7 mM glucose (HG), or in INS‐1 cells cultured at 16.7 mM glucose for 48 h followed by 16.7 mM glucose + 4 mM mannoheptulose (MH reversal) for 48 h. Mannoheptulose was not present during the secretion assay. Insulin secretion is expressed as ng/μg protein. *n* = 3 biologically independent experiments. (g) mRNA levels for the indicated genes in INS‐1 cells cultured at 5 mM glucose (LG), 16.7 mM glucose (HG), or 16.7 mM glucose for 48 h followed by 16.7 mM glucose plus 4 mM mannoheptulose for 48 h (Mh reversal) (*n* = 3 biologically independent experiments). All panels show individual data points and mean ± s.e.m. *p < 0.05, **p < 0.01, ***p < 0.001, ****p < 0.0001. One‐way (b, d, f, g) or two‐way (a, c, e) ANOVA with Bonferroni's multiple comparisons test. For human islet donor details, see Table S1.

Conversely, reducing glycolytic flux by inhibiting glucokinase activity prevented the downregulation of insulin secretion induced by chronic hyperglycaemia (Figure 3). Human islet microtissues were cultured for 14 days at low glucose (5.5 mM), or at high glucose (16.7 mM) with and without 4 mM or 8 mM mannoheptulose, a competitive inhibitor of hexokinases. As previously reported [39], chronic exposure to high glucose enhanced basal insulin secretion at 2.8 mM glucose and inhibited insulin secretion stimulated by 16.7 mM glucose (Figure 3a). These effects were partially (4 mM) or fully (8 mM) prevented by mannoheptulose. This was also the case even when mannoheptulose (4 mM) was included in the assay medium as well as the culture medium. Insulin content was also markedly reduced by chronic hyperglycaemia, a change that was not prevented by 4 mM mannoheptulose but was partially prevented by 8 mM mannoheptulose (Figure 3b).

To determine if the effects of chronic hyperglycaemia were reversible, we cultured human islet microtissues for 7 days at 16.7 mM glucose, followed by 7 days at 16.7 mM glucose with either 4 mM or 8 mM mannoheptulose. Insulin secretion was suppressed by 7 days of high glucose but was partially (4 mM) or fully (8 mM) restored 1 week after the addition of mannoheptulose (Figure 3c). Insulin content, however, was only partially restored (Figure 3d). Similarly, the addition of mannoheptulose for 48 h was able to partially improve glucose‐stimulated insulin secretion but not insulin content in high glucose‐cultured INS‐1 cells (Figure 3e,f). Insulin gene expression was only partially restored (Figure 3g).

**FIGURE 4:**
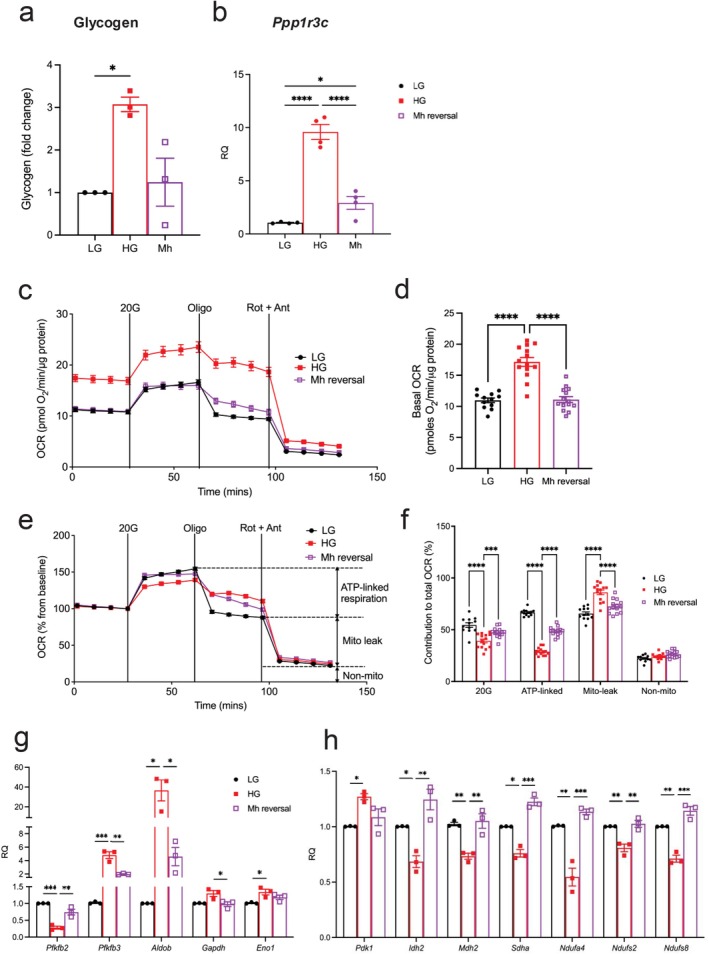
Partial glucokinase inhibition reverses the effects of chronic hyperglycaemia on metabolism. (a, b) Glycogen content (a) (*n* = 3 biologically independent experiments) and mRNA levels of *Ppp1r3c* (b) (*n* = 4 biologically independent experiments) in INS‐1 cells cultured at 5 mM glucose (LG) or 16.7 mM glucose (HG) for 48 h, or at 16.7 mM glucose for 48 h followed by 16.7 mM glucose + 4 mM mannoheptulose for 48 h (Mh reversal). (c) OCR of INS‐1 cells cultured at 5 mM glucose (LG) or 16.7 mM glucose (HG), or of INS‐1 cells cultured at 16.7 mM glucose for 48 h followed by 16.7 mM glucose + 4 mM mannoheptulose (Mh reversal) for 48 h. Mannoheptulose was removed during the assay. OCR was first measured at 2 mM glucose and then after sequential addition of 20 mM glucose (20G), 1 μM oligomycin (Oligo), and 0.5 μM rotenone + 0.5 μM antimycin A (Rot + Ant) (LG, *n* = 12; HG, *n* = 14; Mh reversal, *n* = 14 biologically independent experiments). (d) Basal OCR at 2 mM glucose. Same data as in (c). (e) OCR expressed as the percentage change from baseline (at 2 mM glucose) and after sequential addition of 20 mM glucose (20G), 1 μM oligomycin (Oligo), and 0.5 μM rotenone + 0.5 μM antimycin A (Rot + Ant) (LG, *n* = 12; HG, *n* = 14; Mh reversal, *n* = 14 biologically independent experiments). (f) Percentage change in OCR when glucose was raised from 2 mM to 20 mM (20G), after oligomycin was applied (ATP‐linked), after rotenone and antimycin A were applied (mito‐leak), and remaining non‐mitochondrial OCR (non‐mito). Same data as in (e). (g, h) mRNA levels for the indicated genes in INS‐1 cells cultured at 5 mM glucose (LG), 16.7 mM glucose (HG), or 16.7 mM glucose for 48 h followed by 16.7 mM glucose plus 4 mM mannoheptulose for 48 h (Mh reversal). (g) Glycolytic genes, (h) TCA cycle and electron transport chain genes; *n* = 3 biologically independent experiments. All panels show individual data points and mean ± s.e.m. *p < 0.05, **p < 0.01, ***p < 0.001, ****p < 0.0001. One‐way (a, b, d, g, h) or two‐way (f) ANOVA with Bonferroni's multiple comparisons test.

Previous studies on INS‐1 cells have demonstrated that the ability of partial glucokinase inhibition to *prevent* the effects of chronic hyperglycaemia on insulin secretion is due to changes in expression of insulin and metabolic genes [26]. We now show that glucokinase inhibition, using a dose of mannoheptulose that reduces glucokinase activity to a level equivalent to normoglycaemia, is also largely able to *reverse* the effects of chronic hyperglycaemia on glycogen storage (Figure 4a,b), oxidative metabolism (Figure 4c–f), and metabolic gene expression (Figure 4g,h), thus accounting for the improved insulin secretion.

We further investigated the slow recovery of insulin expression (Figure 3g) by charting the time course of insulin gene expression following the onset or reversal of hyperglycaemia (Figure S6). We quantified the changes in expression of *Ins1, Ins2*, and the insulin gene transcription factors *Mafa* and *Pdx1* up to 72 h following the onset of hyperglycaemia or restoration of euglycaemia. Expression of these genes was very sensitive to a high extracellular glucose concentration, falling by ~75% in the first 12 h following the onset of hyperglycaemia. By contrast, recovery of *Ins1*, *Ins2* and *Mafa* expression following restoration of normoglycaemia was very slow, and was not complete even after 72 h. Although *Pdx1* recovered more quickly, mutagenesis studies of the transcription factor binding sites show the effects of MAFA and PDX1 on insulin promoter activity to be additive [48], suggesting that recovery of the expression of both genes is needed for full restoration of insulin expression.

## 4 Discussion

### 4.1 Effects of Glucokinase Activation

Our results demonstrate conclusively that chronic glucokinase activation at a low extracellular glucose concentration leads to changes in insulin secretion, insulin content, gene expression, glycogen content, and mitochondrial metabolism that resemble those produced by chronic hyperglycaemia. Consistent with these findings, prolonged culture of rat islets at 10 mM glucose with the GKA Ro 280 450 was previously found to mimic glucotoxicity [49], suggesting our conclusions are generalisable across GKAs. Further evidence that enhanced flux through glucokinase is deleterious for beta‐cells comes from mice expressing the severe activating glucokinase mutation Y214C: these mice are hypoglycaemic at birth but rapidly become diabetic because of a loss of beta‐cell functional mass, due to both beta‐cell death and impaired glucose‐stimulated insulin secretion [50].

Our data provide a possible explanation for why glucokinase activators have been unsuccessful in treating T2D in the long term [15, 16, 17, 18, 19, 20]. Although GKAs acutely stimulate insulin secretion, our data show that chronic exposure will cause accumulating changes in beta‐cell gene expression that reduce insulin content and secretion and underlie the long‐term inability of the drug to control glycaemia. The greater their ability to enhance glycolytic flux, the faster the loss of control is likely. Indeed, the ability of MK‐0941 to maximally potentiate insulin secretion at lower glucose concentrations than dorzagliatin may explain the greater propensity of MK‐0941 to cause hypoglycaemia [51]. Thus, glucokinase activation does not seem a viable long‐term strategy for the treatment of T2D.

This raises the question of why patients with activating glucokinase mutations, who initially present with hypoglycaemia, do not ultimately develop diabetes. One possibility is that many GCK‐HI mutations cause relatively mild activation of the enzyme, which may not be sufficient to drive the changes in insulin secretion that predispose to diabetes. On the other hand, partial pancreatectomy has often been used to control glycaemia in patients with severe mutations, such as GCK‐Y214C [49], so that any potential progressive reduction in insulin release due to the mutation cannot be distinguished from that caused by beta‐cell loss.

Future clinical studies should also investigate the impacts of long‐term treatment with glucokinase activators on the liver. Some GKAs, including MK‐0941, have caused hepatosteatosis in T2D trial participants [15]. Mouse models of common human variants of glucokinase regulatory protein (GCKR) reveal that GCKR deficiency results in poorer glycaemic control and raised liver lipids during chronic GKA treatment [52]. Thus, a combination of hepatic and pancreatic effects may contribute to the loss of GKA efficacy.

### 4.2 Effects of Glucokinase Inhibition

We show for the first time that partial glucokinase inhibition can prevent the deleterious effects of chronic hyperglycaemia on human beta‐cells, as it does in mouse islets and INS‐1 cells [26]. We further demonstrate that mannoheptulose is also able to reverse the effects of chronic hyperglycaemia on insulin release, in both human islet microtissues and INS‐1 cells. Restoration of GSIS was apparent whether mannoheptulose was present, as would be the case in vivo, or absent during the assay. Insulin content was only partially restored by mannoheptulose treatment. This is perhaps not surprising given that 4 mM mannoheptulose reduces glucokinase activity at 16.7 mM glucose to the same extent as exposure to 5 mM glucose, and that changes in expression of insulin and key insulin transcription factor genes occur only slowly when euglycaemia is restored.

### 4.3 Strengths and Limitations of the Models

The INS‐1 cell line is widely used in diabetes research and displays appropriate sensitivity to glucotoxicity for this study. However, the model has important limitations, including its rat origin, lower insulin secretion compared to primary islets, excessive proliferation in culture, and incomplete replication of human beta‐cell physiology. The use of human islet microtissues strengthens our findings by closely mimicking native human islet architecture and function while avoiding the drawbacks of native human islet preparations, which tend to suffer from size heterogeneity and rapid functional decline in culture. Microtissues provide a reproducible, homogeneous, and long‐lived system for studying beta‐cell biology and glucose metabolism.

### 4.4 Conclusions

In summary, our results help explain why glucokinase activators ultimately fail to control glycaemia in T2D. Although we focus on the effects of chronic glucokinase activation on beta‐cells, the reported side effects of MK‐0941 and several other GKAs suggest that chronic activation of glucokinase also adversely affects the liver [36]. Our results also show that partial glucokinase inhibition is effective at reducing the adverse effects of chronic hyperglycaemia in human islets, as it does in rodent islets. They further demonstrate that beta‐cell damage induced by hyperglycaemia may be *reversed* by glucokinase inhibition. Thus, our results argue against the use of glucokinase activation to treat diabetes, as this may lead to beta‐cell impairment, and support the view that partial glucokinase inhibition may be a better therapeutic strategy. This strategy could be pursued through development of beta‐cell selective glucokinase inhibitors, akin to hepatoselective GKAs, although we posit that moderate inhibition of liver glucokinase would not be harmful and would likely be beneficial for steatosis [37]. Glucokinase inhibition is therefore a feasible target for preservation of beta‐cell function in T2D.

## Author Contributions

Matthew Lloyd: Methodology, Investigation, Formal analysis, Writing – Reviewing and Editing, Visualization. Natascia Vedovato: Investigation, Formal analysis, Writing – Reviewing and Editing, Visualization. Samuel Mullan: Investigation, Writing – Reviewing and Editing. Alice Elphick: Investigation, Writing – Reviewing and Editing. Aerin Dumenil: Investigation, Writing – Reviewing and Editing. Alexandra C. Title: Methodology, Investigation, Writing – Reviewing and Editing. Burcak Yesildag: Methodology, Investigation, Writing – Reviewing and Editing. Chantal Rufer: Investigation, Writing – Reviewing and Editing. Elizabeth Haythorne: Methodology, Investigation, Formal analysis, Writing – Reviewing and Editing, Visualization, Supervision, Project administration, Funding acquisition. Frances Ashcroft: Conceptualization, Writing – Original draft preparation, Writing – Reviewing and Editing, Supervision, Project administration, Funding acquisition.

## Funding

We thank the UK Medical Research Council (MR/T002107/1 to F.M.A. and E.H.), the Biotechnology and Biological Sciences Research Council (M.L.) and Novo Nordisk (M.L.) for support. M.L. is supported by a Novo Nordisk Postdoctoral Fellowship run in partnership with the University of Oxford and Novo Nordisk, Denmark. E.H. is supported by a University of Edinburgh Chancellor's Fellowship, a British Heart Foundation Research Excellence Award (RE/18/5/34216), a Diabetes Research & Wellness Foundation Pump Priming Grant (WT14175355), a Royal Society Research Grant (RG/R1/251468) and a Royal Society of Edinburgh Small Grant (award ID: 5104). AJD is supported by a British Heart Foundation PhD studentship FS/4yPhD/F/24/34207. Open Access funding was provided by a UKRI block grant to Oxford University.

## Conflicts of Interest

The authors declare no conflicts of interest.

## Supporting information


**Data S1:** Supporting Information.

## Data Availability

The data are available from the authors upon request.
