# Supplementary material for: Contrasting Effects of Chronic Glucokinase Activation and Inhibition on Pancreatic Beta‐Cell Function
Source: FASEB J. 2026 Mar 27;40(7):e71689. doi: 10.1096/fj.202504049R (PMC13023725; doi:10.1096/fj.202504049R)
Supplement: Supplementary file 1 — Data S1: Supporting Information. [file FSB2-40-e71689-s001.pdf]

Supplementary Figure 1

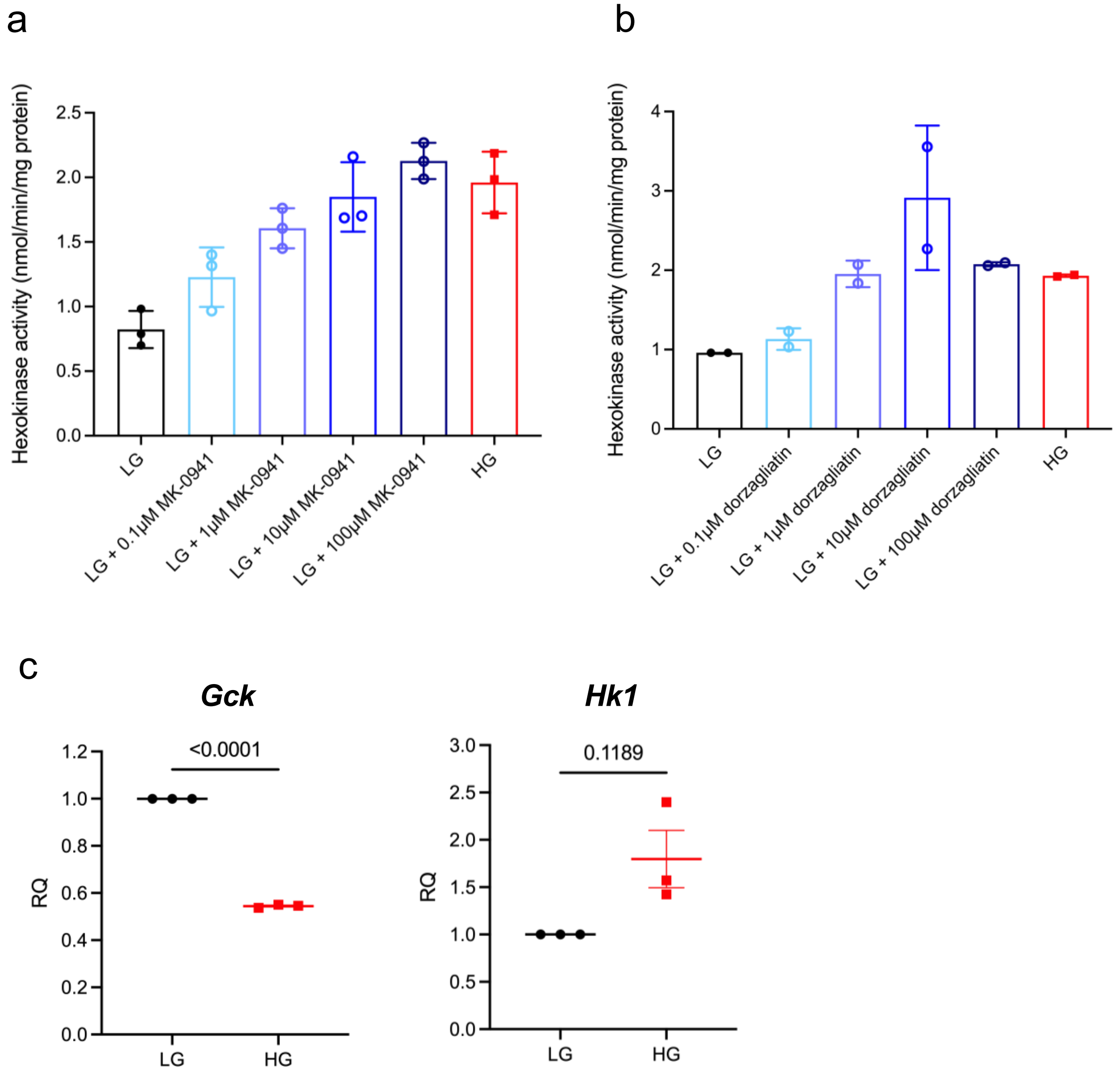

Supplementary Figure 1

(a,b) Hexokinase activity determined in INS-1 cells after 48h incubation at 5m glucose (LG: 5 mM glucose), 5mM glucose plus the indicated concentration of the glucokinase activator (a) MK-0941 or (b) dorzagliatin, or at high glucose (HG, 25mM glucose). N=1 experiment with (a) 3 or (b) 2 biological replicates.
(c) Relative mRNA expression of *Gck* and *Hk1* measured by qPCR in INS-1 cells cultured at 5mM glucose (LG) or 25mM glucose (HG) for 48h (n=3 biologically independent experiments. Individual data points are shown. Mean±s.e.m. unpaired t-test, p values given above the plot) .

Supplementary Figure 2

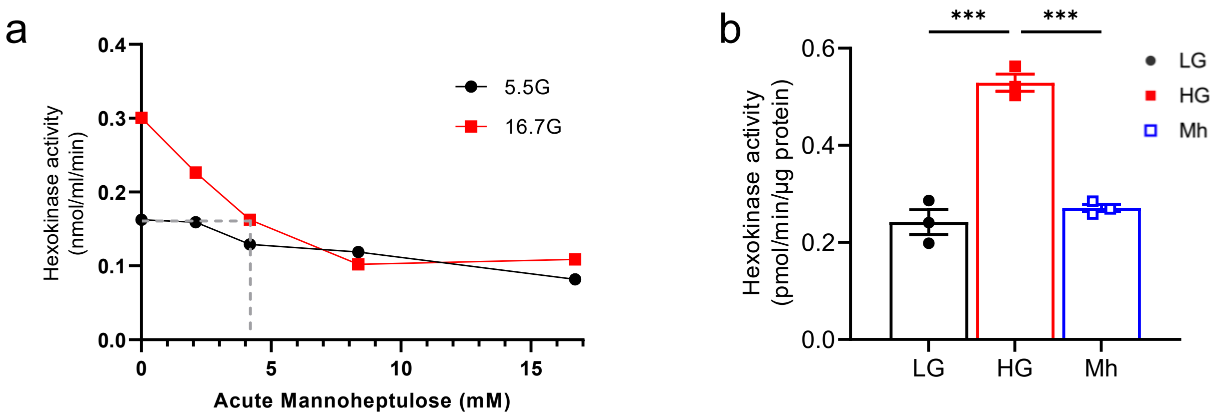

Supplementary Figure 2

(a) Hexokinase activity measured acutely in INS-1 cells at 5.5mM or 16.7mM glucose plus the indicated concentration of the glucokinase inhibitor mannoheptulose (n=1 biologically independent experiment). Cells were cultured at 11mM glucose.

(b) Hexokinase activity measured in INS-1 cells cultured for 48h at 5.5mM glucose (LG), 16.7mM glucose (HG) or 16.7mM glucose + 4mM mannoheptulose (Mh). (n=3 biologically independent experiments). Activity is normalised to protein concentration to account for differences in cell number. Individual data points and mean ± s.e.m are shown. ***p<0.01. One way ANOVA.

Legends to Supplementary Figures 3-5

Effect of glucokinase activation and inhibition on insulin secretion in human islets

Supplementary Figure 3 (donor 229)

(a,b) Insulin secretion at 2.8mM and 16.7mM glucose (a) and insulin content (b) in human islet microtissues cultured at 5.5mM glucose, 5.5mM glucose + MK-0941 (0.1, 1, or 10 μM), and 16.7mmM glucose for 14 days.

(c,d) Insulin secretion at 2.8mM and 16.7mM glucose (a) and insulin content (b) in human islet microtissues cultured for 14 days at 5.5mM glucose, 16.7mM glucose, and 16.7mmM glucose + 4mM or 8mM mannoheptulose. The drug was removed from the assay medium for all experiments except for the data shown in olive bars (4mM mannoheptulose).

(e,f) Insulin secretion at 2.8mM and 16.7mM glucose (a) and insulin content (b) in human islet microtissues cultured for 14 days at 5.5mM glucose or 16.7mM glucose, for 7days at 5mM glucose followed by 7 days at 16.7mM glucose, or for 7 days at 16.7mmM glucose followed by 7 days at 16.7 mM glucose + either 4mM or 8mM mannoheptulose. The drug was removed from the assay medium for all experiments.

Data were obtained from islets isolated from a donor without diabetes (donor 229; for details see Supplementary Table 1).

Individual data points and mean ± s.e.m (n=6 replicates). *P<0.05, **P < 0.01, ***P < 0.001

Supplementary Figure 4 (donor 230)

Legend as in Supplementary Fig 3 but for donor 199.

(a,b) GKA = 0.1μM MK-0941

Supplementary Figure 5 (donor 199)

Legend as in Supplementary Fig 3 but for donor 199.

(a,b) GKA = 0.1μM MK-0941

Supplementary Figure 3 – Donor 229

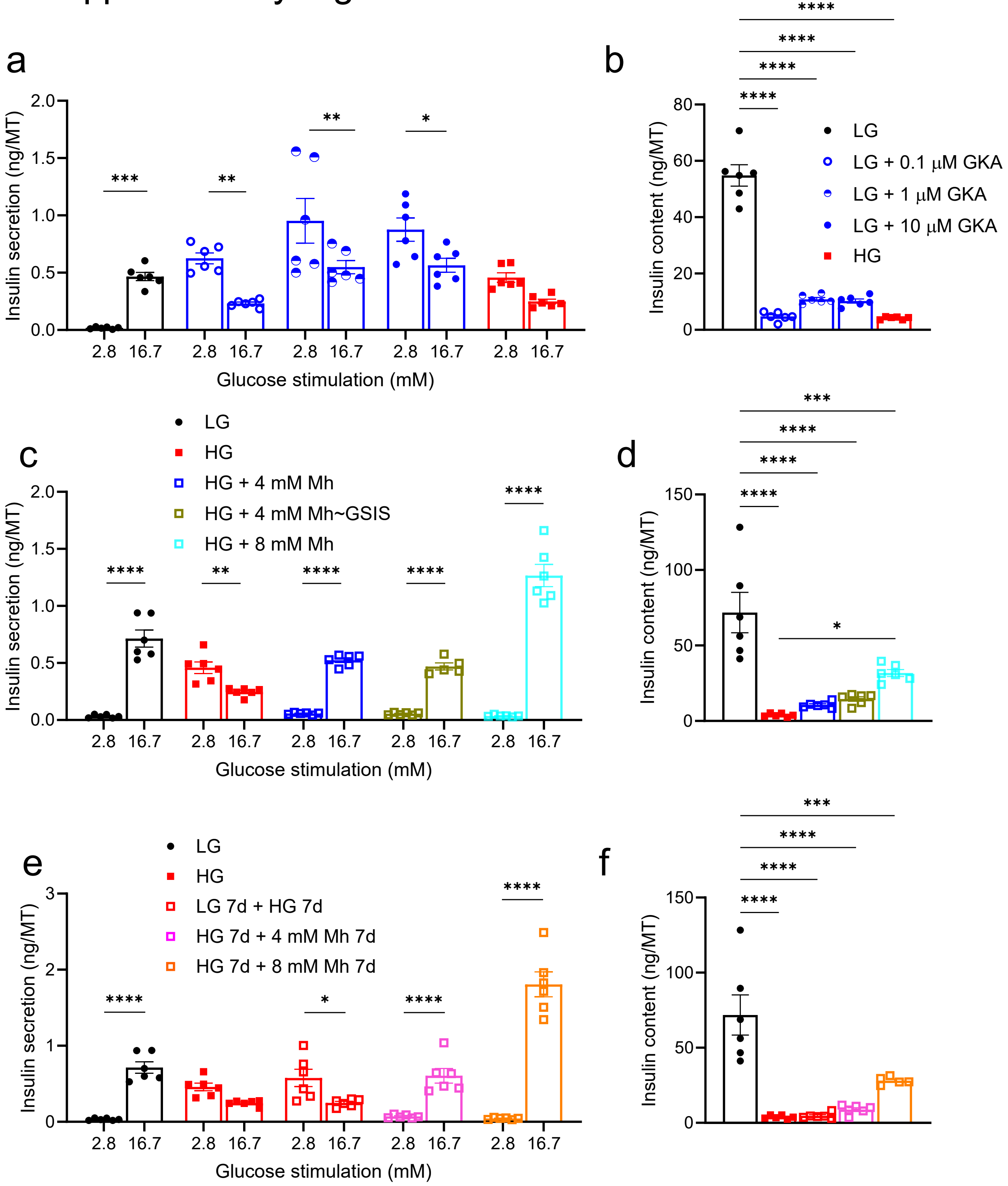

Supplementary Figure 4 - Donor 230

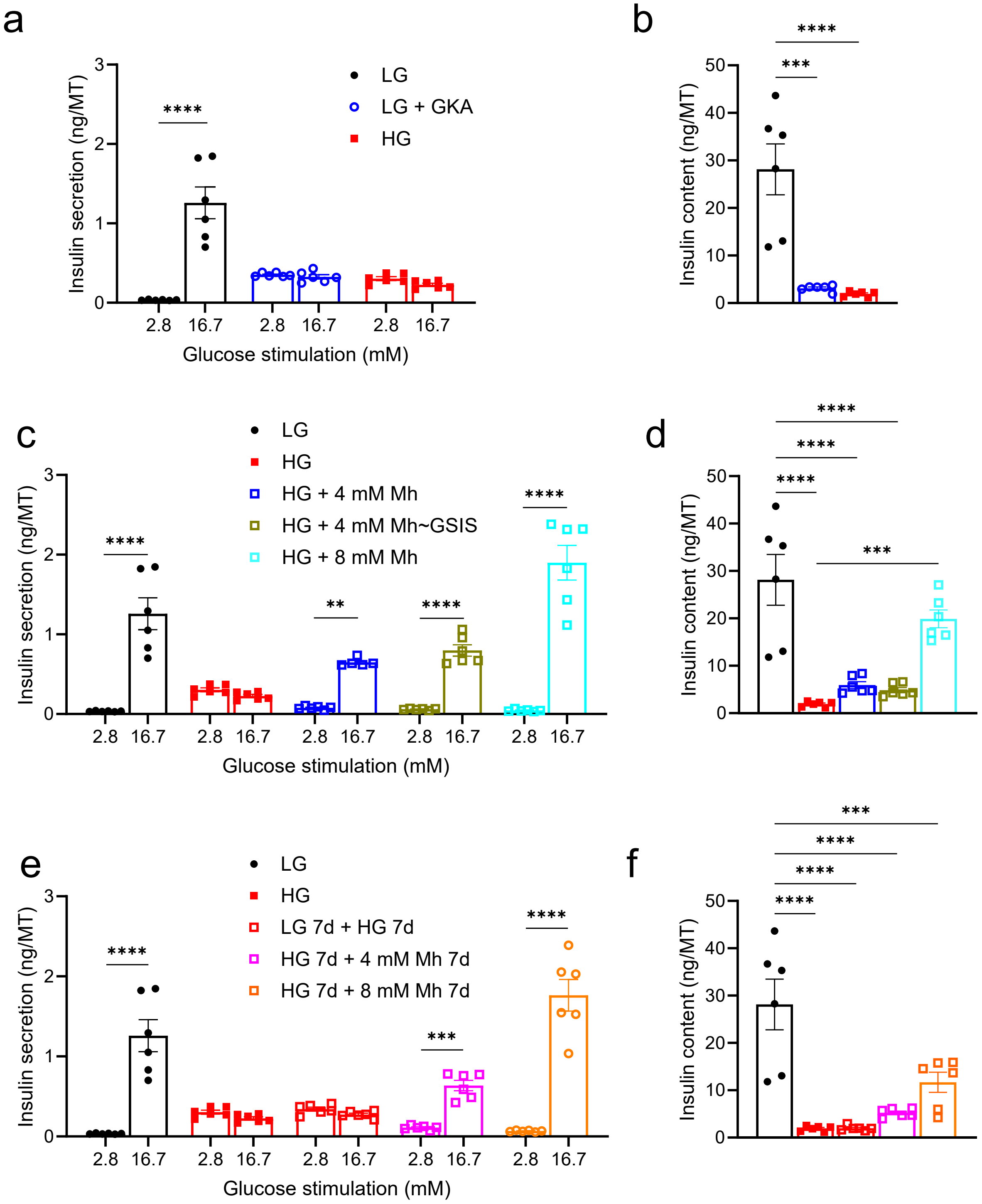

Supplementary Figure 5 – Donor199

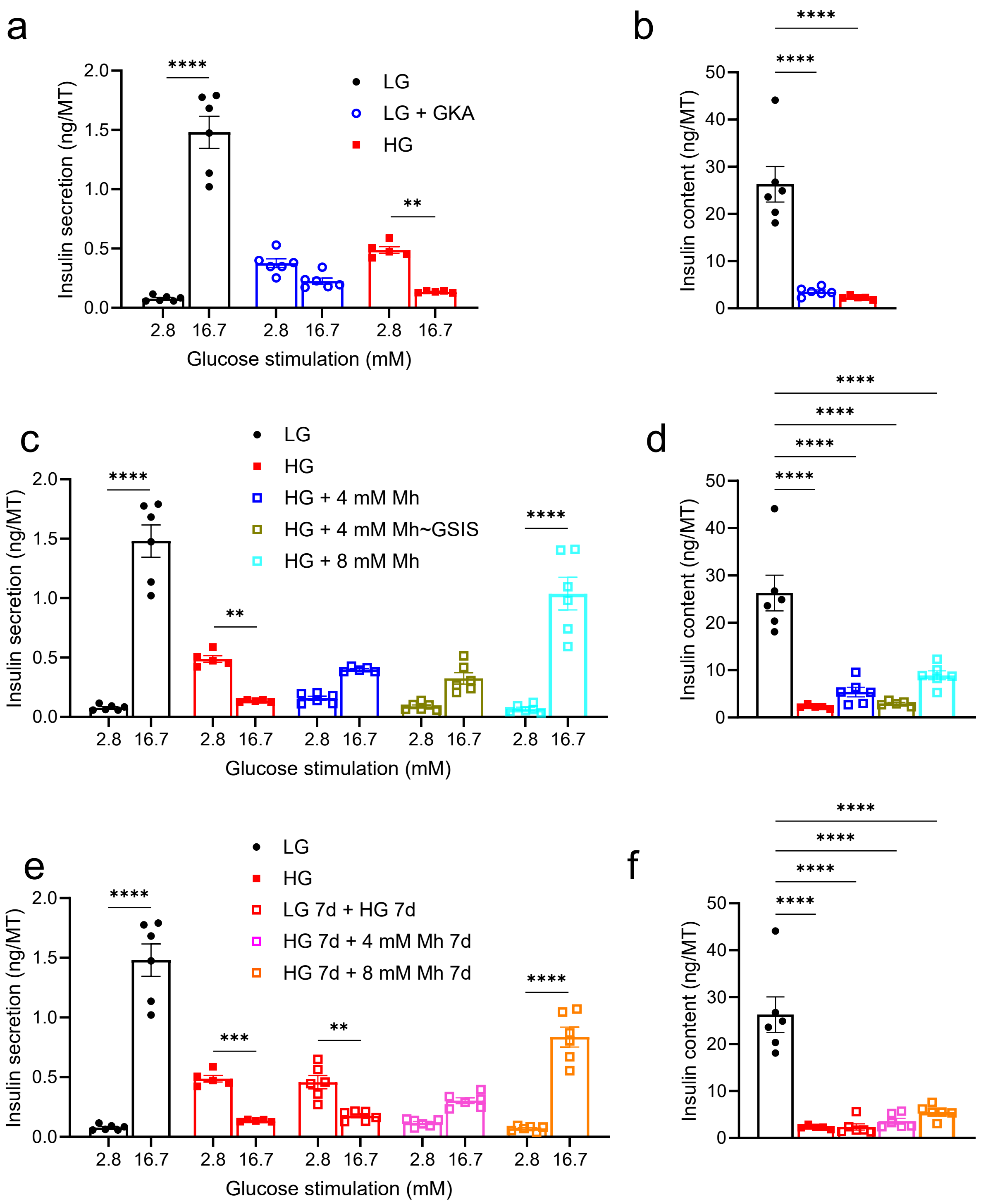

Supplementary Figure 6

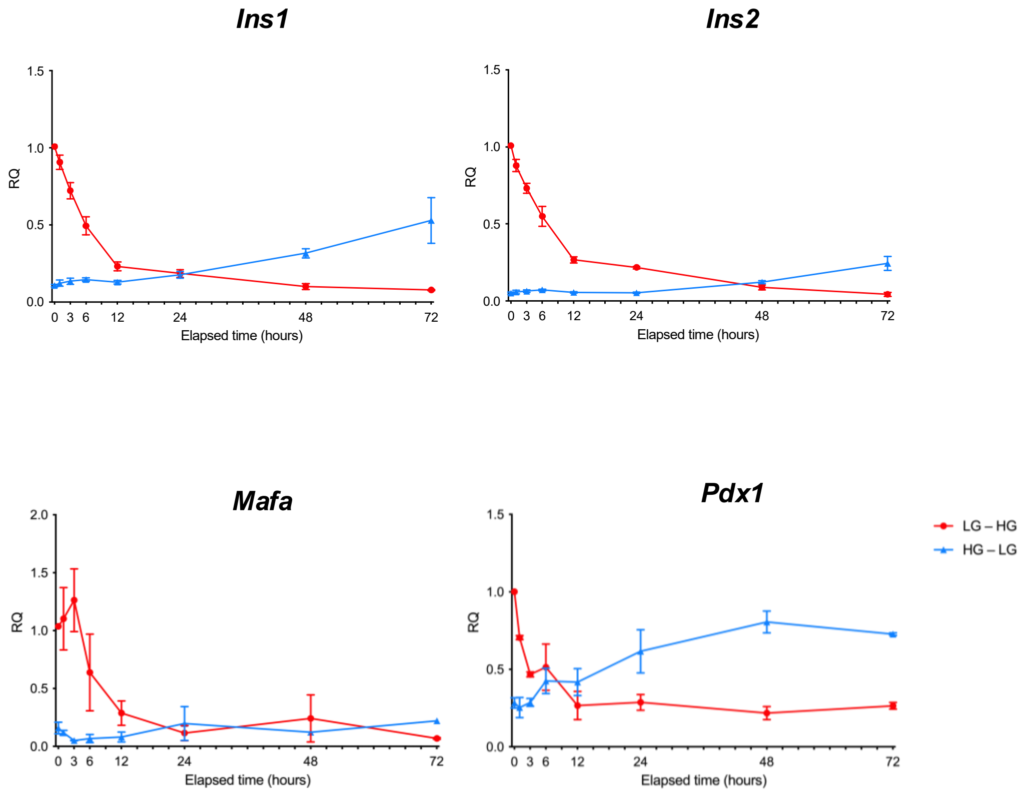

Supplementary Figure 6: Time course of mRNA levels of insulin genes, *Ins1* and *Ins2,* and β-cell identity genes, *Mafa* and *Pdx1,* following the onset or reversal of hyperglycaemia in INS-1 cells. Samples were collected at time points of 1, 3, 6, 12, 24, 48, and 72 hours following the onset (red) or reversal (blue) of hyperglycaemia. Cells were pre-cultured at 5mM (low glucose: LG) or 25 mM glucose (high glucose: HG) for 48h before the media was changed to the opposite condition at the time point 0 (mean ±s.e.m of n=3 biologically independent experiments).

Supplementary Table 1

Donor	1	2	3	4
**Donor ID**	230	229	199	130
**UNOS ID**	AKJG366	AKIK131	AJFT363	AGJT231
**Donor age (years)**	58	39	51	62
**Donor sex (M/F)**	F	M	M	M
**Donor BMI (kg/m^2)**	30.95	26.9	30.05	31.25
**Donor HbA$_{1c}$**	4.7%	5.2%	5.3%	5.5%
**Donor history of diabetes?**	No	No	No	No

Human donor characteristics.
Donors 1-3 were used for the insulin secretion studies. Donor 4 was used for transcriptomic analysis.

Supplementary Table 2

a

List of Taqman probes

Gene	Catalogue number (Applied Biosystems)
Aldob	Rn01768292_m1
Eno1	Rn01518942_gH
Gapdh	Rn01775763_g1
Hprt1	Rn01527840_m1
Hspa8	Rn00821191_g1
Idh2	Rn01478119_m1
Ins1	Rn02121433_g1
Ins2	Mm00731595_gH
Mafa	Rn00845206_s1
Mdh2	Mm00725890_s1
Ndufa4	Mm00809672_s1
Ndufs2	Rn01411711_m1
Ndufs8	Mm00523063_m1
Neurod1	Rn01280117_m1
Nkx6-1	Rn01450076_m1
Pax6	Rn00689608_m1
Pdk1	Rn00587598_m1
Pdx1	Rn00755591_m1
Pfkfb2	Mm00435575_m1
Pfkfb3	Rn00678825_m1
Ppp1r3c	Mm01204084_m1
Sdha	Rn00590475_m1
Txnip	Mm00452393_m1

b

Custom primer sequences

Gene	Forward primer	Reverse primer
Actb	5'-CCCGCGAGTACAACCTTCTTG-3'	5'-GTCATCCATGGCGAACTGGTG-3'
Hprt1	5'-CTCATGGACTGATTATGGACAGGAC-3'	5'-GCAGGTCAGCAAAGAACTTATAGCC-3'
Gck	5'-AATCTTGCGGAACACTGAG-3'	5'-CCACATTCTGCATTTCCTC-3'
Hk1	5'-GTAGAACTCACCCCAGTCCC-3'	5'-GGGTCTCCTGATCCTTGGGAG-3'

Supplementary Table 3

Expression of hexokinase isoforms in human islets under low glucose and chronic hyperglycaemia culture conditions (RNA Sequencing using TempO-Seq technology)

	Low glucose (5.5 mM glucose)		Chronic hyperglycaemia (16.7mM glucose)	
**Gene**	CPMs	fold difference (GSK/gene)	CPMs	fold difference (GSK/gene)
HK1	1.02	56.6	0.74	55.7
HK2	0.66	88.4	0.66	62.1
HK3	0.38	154.2	0.08	501.2
GCK	57.98	1.0	41.10	1.0
